# Retraction Note: Investigating the use of green synthesized copper oxide nanoparticles from Melia azedarach to combat cadmium stress in wheat

**DOI:** 10.1038/s41598-026-54940-z

**Published:** 2026-05-26

**Authors:** Iram Naz, Maryam, Aysha Arif Chahel, Ishtiaq Ahmad, Amber Raza, Muhammad Waseem Haider, Muhammad Saqlain Zaheer, Muhammad Waheed Riaz, Muhammad Rizwan, Salim Manoharadas

**Affiliations:** 1 Department of Botany, The Government Sadiq College Women University, Bahawalpur, Pakistan; 2 Department of Horticultural Sciences, Faculty of Agriculture and Environment, Islamia University of Bahawalpur, Bahawalpur, 63100 Pakistan; 3 Department of Agricultural Engineering, Khwaja Fareed University of Engineering and Information Technology, Rahim Yar Khan, Pakistan; 4 State Key Laboratory of Wheat Breeding, Group of Wheat Quality and Molecular Breeding, College of Agronomy, Shandong Agricultural University, Tai’an, 271000 Shandong China; 5 Institute of Crop Science and Resource Conservation (INRES), University of Bonn, 53115 Bonn, Germany; 6 Department of Botany and Microbiology, College of Science, King Saud University, 11451 Riyadh, Saudi Arabia

Retraction to: *Scientific Reports* 10.1038/s41598-025-10168-x, published online 25 July 2025.

The Editors have retracted this Article.

After publication, concerns have been raised about the validity of the data in some of the figures, in particular Figs. 4 and 5, as well as apparent overlaps between images representing different conditions in Table 1. These concerns were corroborated by the Editors. The data was not requested, since the nature of the concerns around Fig. 4 and 5 cannot be resolved with data. The Editors have therefore lost confidence in the integrity of this Article.

The Authors disagree with this retraction.

